# Corneal Lymphatics: Role in Ocular Inflammation as Inducer and Responder of Adaptive Immunity

**DOI:** 10.4172/2155-9899.1000256

**Published:** 2014-09-27

**Authors:** Sunil K. Chauhan, Thomas H. Dohlman, Reza Dana

**Affiliations:** Schepens Eye Research Institute and Massachusetts Eye and Ear Infirmary, Harvard Medical School, Boston, Massachusetts 02114, USA

**Keywords:** Cornea, Lymphangiogenesis, Adaptive immunity, Ocular inflammation

## Abstract

The normal cornea is devoid of lymphatic and blood vessels, thus suppressing both the afferent (lymphatic) and efferent (vascular) arms of the immune response–contributing to its ‘immune privilege’. Inflammation, however, negates this unique ‘immune’ and ‘angiogenic’ privilege of the cornea. Abnormal blood vessel growth from pre-existing limbal vessels into the cornea has been studied for many years, but it is only recently that the significance of new lymphatic vessels (lymphangiogenesis) in ocular inflammatory diseases has been demonstrated. Whereas blood vessels in inflamed ocular surface provide a route of entry for immune effector cells to the cornea, lymphatics facilitate the exit of antigen-presenting cells and antigenic material from the cornea to regional lymph nodes, thus promoting induction of adaptive immune response. This review summarizes the current evidence for lymphangiogenesis in the cornea, and describes its molecular mediators; and discusses the interface between corneal lymphangiogenesis and adaptive immunity. Furthermore, the pathophysiologic implications of corneal lymphangiogenesis in the setting of allo- and autoimmune-mediated corneal inflammation are discussed.

## Introduction

The lymphatic vasculature plays an important role in the maintenance of fluid homeostasis, regulation of lipids, and trafficking of antigen-presenting cells from peripheral tissue to regional draining lymph nodes. In contrast to other tissues, the cornea is unique in that it actively maintains an avascular and alymphatic state, which limits antigen-presentation and leads to its status as an immune privileged tissue. However, the cornea’s immune privilege can be disrupted, and blood as well as lymphatic vessels can develop in a number of pathologic conditions [1].

**Figure 1:**
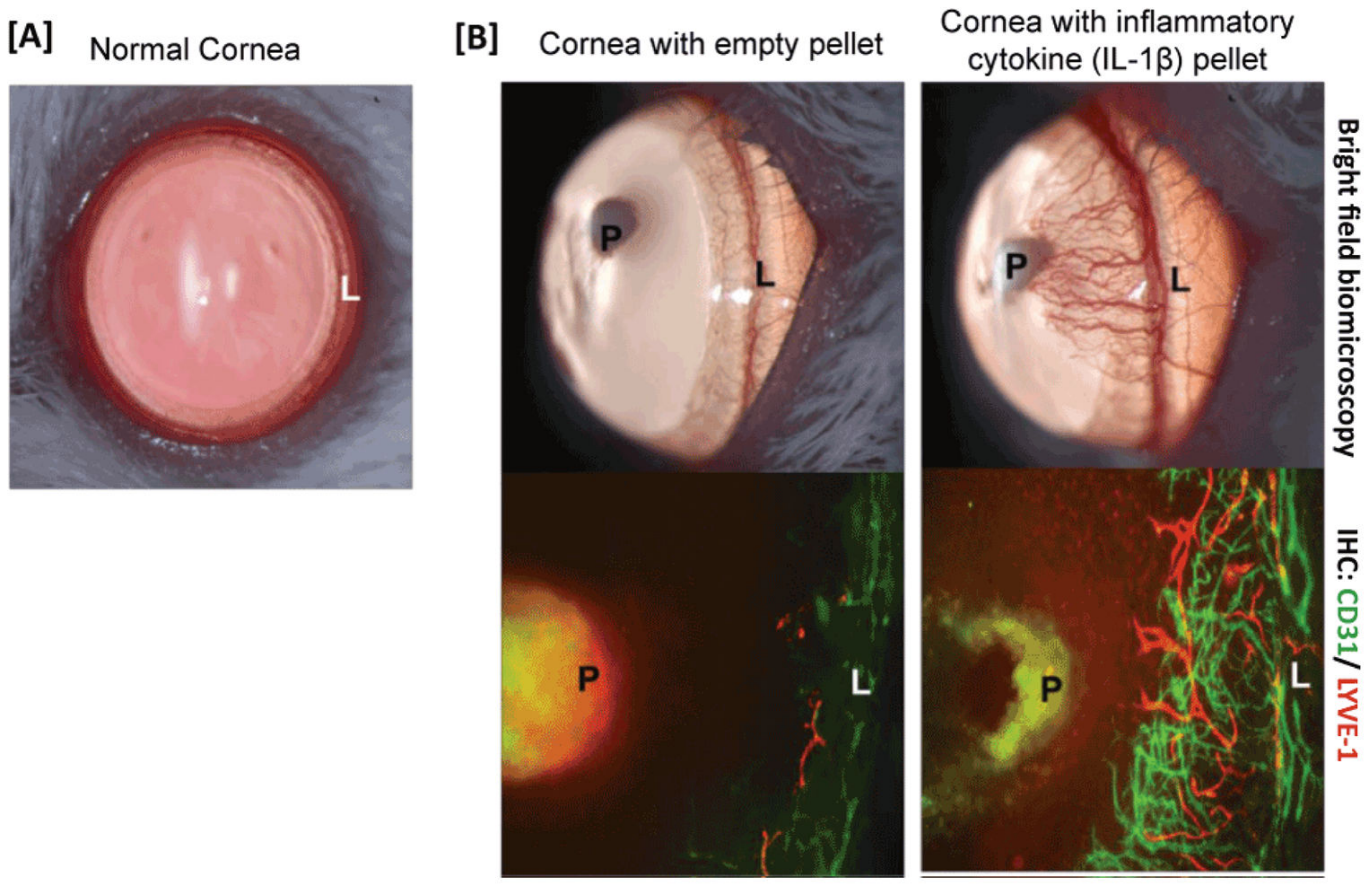
Angiogenic privilege of cornea. [A] Normal cornea is a blood and lymphatic vessel-free tissue. Vessels are restricted only to the periphery of the cornea (limbal area). [B] In response to inflammation, this “angiogenic” privilege of the cornea can be lost leading to the ingrowth of both blood and lymphatic vessels. Micrographs from a mouse corneal micropocket model of neovascularization show that pellets containing the inflammatory cytokine IL-1β robustly induce ingrowth of both new blood (CD31^+^LYVE1^−^) and lymphatic (LYVE1^+^CD31^lo^) vessels. (L: Limbus; P: Pellet).

The cornea is circumferentially surrounded by lymphatic vessels located in the limbus [2]. These vessels connect to the conjunctival lymphatic network but, under homeostatic conditions, do not enter the cornea (Figure 1A). Under inflammatory conditions, however, these lymphatic vessels can give rise to new lymphatics, which do extend into the cornea (Figure 1B) [3]. Blood and lymphatic vessel formation, as in other tissues, is primarily mediated by the vascular endothelial growth factor (VEGF) family, with lymphatic vessel formation specifically coordinated by the interactions of VEGF Receptor-3 with its ligands, VEGF-C and VEGF-D [4]. Activation of VEGFR-3 by VEGF-C leads to phosphorylation of protein kinase B (AKT) and extracellular signal-regulated kinase (ERK), which in turn leads to lymphatic endothelial cell proliferation and lymphatic tube formation [5,6]. In addition to VEGF-C/D signaling through VEGFR-3, additional factors known to promote lymphangiogenesis include angiopoietin-2 and integrin alpha 5. Studies inhibiting both type of factors have demonstrated efficacy in preferentially blocking corneal lymphangiogenesis versus hemangiogenesis [7–9].

Corneal lymphatic vessels may also be able to develop de novo, independent of limbal lymphatics. Under inflammatory conditions CD11b+ macrophages in the corneal stroma have been observed to express the ‘classic’ lymphangiogenic markers Lymphatic Vessel Endothelial Receptor 1 (LYVE-1) and Prospero homeobox 1 (PROX-1). Interestingly, *in vitro*, macrophages are able to aggregate into tube-like structures, which express the lymphatic markers LYVE-1 and podoplanin, suggesting a direct contribution of these cells to lymphatic vessels. Further, macrophages contribute to the maintenance of corneal lymphatics in inflammation [10]. The majority of the lymphatic vasculature develops from homeobox gene PROX-1–expressing cells of the venous circulation during embryogenesis, which then undergo further remodeling [11]. PROX-1 expression is critical for the initial formation of lymphatic endothelial cells from venous cells [12]. Lymphatic endothelial cells interlock in two distinct patterns to form lymphatic capillaries in peripheral tissue. Lymphatic endothelial cells in initial lymphatics are connected by discontinuous adhesion proteins called “buttons”, which are able to create openings through which fluid and immune cells can enter. Lymphatic endothelial cells in collecting lymphatics are continuously attached in a “zipper”-like fashion [13,14].

In this review we aim to (i) describe the evidence of corneal lymphangiogenesis, (ii) outline its molecular mediators, and the interaction between corneal lymphangiogenesis and adaptive immunity, and (iii) discuss the pathophysiologic implications of corneal lymphangiogenesis in the setting of allo- and autoimmune-mediated corneal inflammation.

## Corneal Angiogenic Privilege

A pristine visual axis is of the utmost importance to preserve proper visual function [15]. Accordingly, under homeostatic conditions, the cornea is both alymphatic and avascular. The importance of this “angiogenic privilege” is emphasized by the presence of multiple mechanisms that serve to maintain it.

One mechanism that helps to keep the cornea alymphatic and avascular is through controlling the expression and availability of VEGF receptors and ligands, which is accomplished through the expression of several anti-angiogenic factors. The corneal epithelium expresses a soluble form of VEGFR-3, which acts as a decoy receptor and binds VEGF-C and VEGF-D [5,16], as well as soluble VEGFR-2, which prevents lymphatic invasion of the central cornea [17]. The cornea also expresses thrombospondin-1, which is known to regulate the production of VEGF-C by monocytes/macrophages through binding of CD36 [18]. The corneal epithelium additionally expresses soluble VEGFR1, which limits hemangiogenesis by acting as a decoy receptor for VEGF-A [19]. Other anti-angiogenic factors known to be expressed by the cornea include Pigment epithelium-derived factor (PEDF), a potent inhibitor expressed by the corneal epithelium [20,21] as well as angiostatin, which is also expressed by the corneal epithelium and is capable of binding multiple targets on vascular endothelial cells to inhibit proliferation and migration [22–26]. The corneal epithelium also expresses endostatin, which can inhibit angiogenesis by directly inhibiting vascular endothelial cell proliferation or by blocking VEGFR-2 signaling [27,28].

The avascular/alymphatic (angiogenic privilege) state of the cornea is intricately linked to its immune privileged state, which is maintained among other factors by the expression of a range of immunoregulatory factors [29–31]. These anti-inflammatory factors in turn contribute to immune and angiogenic privilege by regulating the functional interface between immune and vascular endothelial cells. For example, Transforming growth factor (TGF)-β2 regulates dendritic cell maturation [32] and the corneal epithelium expresses Fas ligand (FasL), which induces apoptosis of FAS-expressing immune cells [33]. Fas is also expressed by neovessels arising under inflammatory conditions, but not quiescent vessels, meaning that Fas/FasL interactions also have a direct anti-angiogenic effect [34]. Programmed death-ligand 1 (PDL-1) is another regulatory molecule expressed constitutively at high levels by the corneal epithelium that downregulates T cell responses and induces immune cell apoptosis [32, 35]. PDL-1 is also expressed by vascular endothelial cells and its inhibition leads to a significant increase in cell proliferation [36].

Additional factors contributing to corneal immune privilege include the general anti-inflammatory proteins Interleukin (IL)-10 [37] and IL-1Ra. IL-1Ra inhibits pro-inflammatory IL-1α and IL-1 β by competitively binding IL-1R without inducing signaling [38]. By down-regulating inflammation these factors preserve expression of the corneal epithelium derived anti-angiogenic factors and prevent the recruitment and activation of immune cells such as neutrophils, macrophages, and T cells, all of which promote heme- and lymphangiogenesis by secreting VEGF ligands [39–42].

## Mechanisms of Corneal Lymphangiogenesis

Loss of corneal angiogenic privilege is due to a deficit in the cornea’s anti-angiogenic factors relative to pro-angiogenic factors, and is evident in a number of pathologic conditions, including trauma, infection, corneal transplant rejection, and dry eye disease [1,43]. Although the inciting injury or disease may vary, the formation of lymphatic vessels allows the host to accomplish the same task, namely controlling tissue injury and inflammation by facilitating antigen-presenting cell trafficking to regional lymphoid tissues where T cell responses can be orchestrated. In contrast, lymphangiogenesis may also directly contribute to the resolution of inflammation by clearing cells and debris generated as a result of inflammation-induced tissue injury [44,45]. Indeed, in some situations anti-lymphangiogenic therapy has been observed to actually delay the resolution of inflammation [46].

Injury to the cornea, including mechanical trauma and chemical burn, generates a non-specific inflammatory response driven by the innate immune system. As part of this response, damaged corneal tissue produces pro-inflammatory factors, including Tumor necrosis factor (TNF)-α, IL-1, chemokines such as CC chemokine ligand (CCL) 2 and CCL20, and integrins such as Intercellular Adhesion Molecule 1 (ICAM-1) [40,47–49]. These factors not only damage the ocular surface, but also facilitate the recruitment of innate immune cells including neutrophils and macrophages, which promote lymphangiogenesis through production of VEGF-C and VEGF-D [39,42,50].

Pathologic blood and lymphatic vessels are also commonly seen in infectious processes such as herpes simplex viral (HSV) keratitis. Lymphatic vessel formation in HSV infected corneas is stimulated by the contributions of the general pro-inflammatory factors described above, as well as by the virus itself, which stimulates corneal epithelial cells to drive lymphangiogenesis through VEGF-A/VEGFR-2 signaling [51]. In addition, CD8^+^ T cells generated as part of the adaptive immune response to HSV also contribute to lymphangiogenesis through their production of VEGF-C [52].

Along with infection, corneal transplant rejection and dry eye disease are two other common conditions in which lymphangiogenesis is present and plays an important role. Corneal transplantation is the most common form of solid tissue transplantation, with over 100,000 transplants performed globally each year (as published by the Eye Bank Association of America-EBAA). Dry eye disease is a chronic, immunoinflammatory disorder of the ocular surface, which affects millions of individuals [53,54]. In both corneal transplantation and dry eye disease, lymphangiogenesis permits APCs to reach the draining lymph nodes where they then prime naïve T cells and induce a T-helper response. These T cells then traffic back to the cornea where they mediate graft rejection or drive the chronic ocular surface inflammation seen in dry eye disease.

## Corneal Lymphatics as Inducers of Adaptive Immunity

One of the major functions of the lymphatic vasculature in all tissues is to facilitate adaptive immunity. Lymphatic vessels are crucial to the host’s ability to respond to infectious agents and target antigens, as they facilitate the trafficking of antigen-presenting cells (APCs) to draining lymph nodes where they present antigen to naïve T cells. In the case of most microbial infections, induction of an adaptive immune response is crucial to the host’s ability to defend itself. However, in some cases, adaptive immunity may not be as beneficial, as is seen in autoimmune conditions such as multiple sclerosis, rheumatoid arthritis, and dry eye disease [53,55,56]. In these conditions, an adaptive immune response is inappropriately mounted, leading to destruction of normal tissue and chronic inflammation. The adaptive immune response can be similarly detrimental in the setting of organ transplantation, as the host immune system sees transplanted tissue as foreign, leading to induction of allo-reactive T cells and subsequent rejection of the transplant.

**Figure 2:**
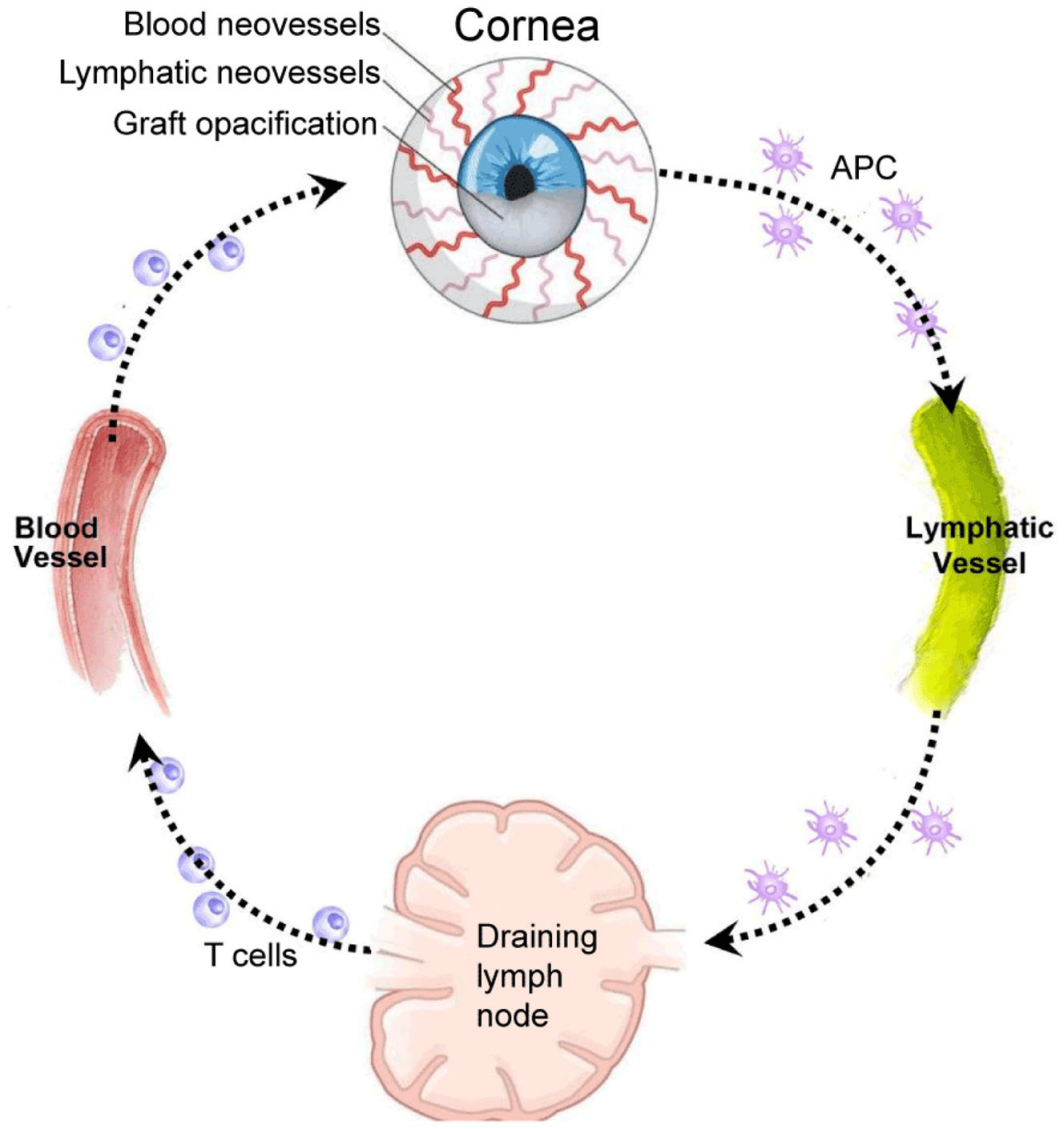
Function of lymphatic and blood vessels in corneal alloimmunity. Lymphatics (afferent arm of immune system) transport antigens and antigen-presenting cells (APCs) from the graft site to the draining lymph nodes, where T cells are primed and expanded. Alloreactive T cells return to the cornea via blood vessels (efferent arm) and mediate transplant rejection.

The alloimmune response can be visualized as consisting of afferent and efferent arms (Figure 2). Lymphatic vessels mediate the afferent arm of the immune system by facilitating the migration of APCs and alloantigens to the draining lymph nodes, where they prime alloreactive effector T cells, which then migrate back to the graft via blood vessels (the efferent arm). The afferent arm begins with functional changes in lymphatic endothelial cells and APCs in response to inflammatory cytokines. Due to inflammation immature APCs adopt a mature phenotype, which includes upregulation of major histocompatibility complex II and costimulatory molecules [57], as well as downregulation of the chemokine receptors CC chemokine receptor (CCR)1, CCR2, CCR5, and chemokine (C-X-C motif) receptor 1 (CXCR1) as well as upregulation of CCR7 [58,59]. These APCs then enter lymphatic capillaries, a process that relies on CCR7^+^ APCs following a CCL21 gradient, as well as the interplay of ICAM-1 and vascular cell adhesion molecule 1 (VCAM-1) and their ligands [60–63]. Upon reaching the parafollicular cortex, APCs then present antigen to naïve T cells, which differentiate into CD4^+^ T cells, the predominant effector cells in corneal transplantation [64,65].

The importance of lymphatic vessels in alloimmunity is illustrated by the high rate of rejection seen in those transplants performed in corneal beds with pre-existing lymphatics, so called ‘high-risk’ hosts [66–68]. In these hosts, with a luxurious supply of lymphatics in the recipient graft bed, APC trafficking and allo-sensitization is significantly increased compared to low-risk hosts with an avascular cornea, and can begin almost immediately; APCs are detectable in the draining lymph nodes at 4 hours after transplantation [69], and peak at 24 hours post transplantation [70]. Further, the effector T cell response is significantly increased in ‘high-risk’ hosts up to 72 hours post transplantation [71]. Several anti-lymphangiogenic therapies have shown promise in improving corneal transplant survival, including the neutralization of VEGF-C [72] and using a VEGFR-3 trap [73]. Anti-lymphatic therapy has also been used to improve high-risk corneal transplant outcomes [67]. In this study, high-risk host beds were treated with anti-lymphatics prior to transplantation in order to reduce lymphatic vessel density. These studies further highlight the importance of lymphangiogenesis in alloimmunity, although the utility of anti-lymphatic therapy in the clinical setting remains to be seen.

## Corneal Lymphatics as Responders of Adaptive Immunity

The formation of new lymphatic vessels is a dynamic process during embryogenesis but is relatively rare and selectively regulated in adulthood. Inflammation is the main physiologic event that evokes formation of new lymphatic vessels in adulthood [74,75]. It is generally recognized that early innate immune responses play a crucial role in the induction of lymphangiogenesis in most corneal inflammatory disorders, including transplant rejection and infection, which in turn facilitate migration of APCs toward the draining lymphoid tissues to prime naïve T cells leading to an adaptive immune response. Innate inflammation triggers lymphangiogenesis via early surge of pro-inflammatory molecules that orchestrates the inflammatory response, which in turn upregulates expression of vascular growth factors and cytokines promoting survival, migration, and proliferation of lymphatic endothelial cells [76–78]. However, recent studies have demonstrated that not only innate inflammatory responses, but also late adaptive immune responses, can play a critical role in the induction of lymphangiogenesis in chronic inflammatory conditions such as malignancy and autoimmune disorders [79,80].

**Figure 3:**
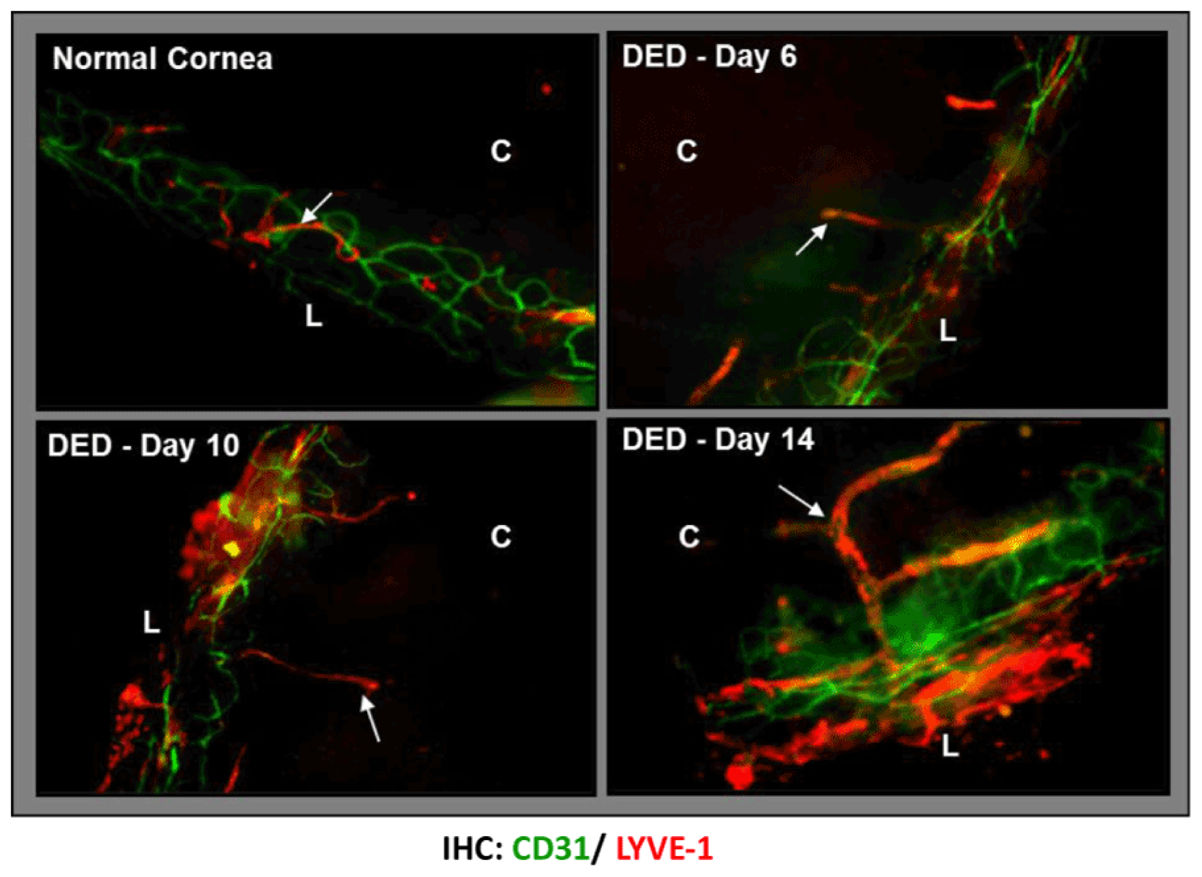
Selective ingrowth of corneal lymphatic vessels in dry eye disease. Confocal micrographs showing corneal lymphangiogenesis in normal cornea and in dry eye corneas at days 6, 10, and 14 post-induction of dry eye in a mouse model of dry eye disease. The lymphatic vessels (LYVE1^+^CD31^lo^) increase both in area and caliber, and grow towards the cornea center with disease progression. The lymphatics are unaccompanied by blood vessels (CD31^hi^/LYVE1^−^). (Lymphatics marked by arrows; C: Cornea; L: Limbus). Adapted from [81].

In a model of T-helper-17 cell (Th17)-dominant autoimmune dry eye disease, we have recently reported the selective occurrence of corneal lymphangiogenesis and significantly elevated homing of mature APCs to the lymphoid tissues where they induce autoreactive IL17^+^CD4^+^T cell (Th17) responses [81,82]. Dry eye disease, which is the most common ophthalmic pathological condition, is a complex, multifactorial, immune-mediated disorder in which chronic ocular surface inflammation is sustained by ongoing activation and infiltration of pathogenic immune cells, primarily Th17 cells [83]. We have demonstrated that lymphangiogenesis, without concurrent growth of blood vessels (hemangiogenesis), occurs in the cornea of mice with dry eyes [81]. Interestingly, along with the progression of the disease, these lymphatics not only grow toward the central cornea, but also show significantly increased caliber compared to those restricted to the limbal areas of normal corneas (Figure 3). This is in contrast to other robust models of corneal inflammation such as transplantation or infection where there is either parallel outgrowth of blood and lymphatic vessels, or the blood vessels are precedent over the lymphatics [66–68]. New ingrowth of lymphatics in dry eye corneas not only provides a link between chronic ocular surface inflammation and the generation of T cell mediated immunity in the lymphoid compartment, but also offers an example of how lymphangiogenesis and hemangiogenesis can be ‘naturally’ dissociated in a pathological state.

**Figure 4:**
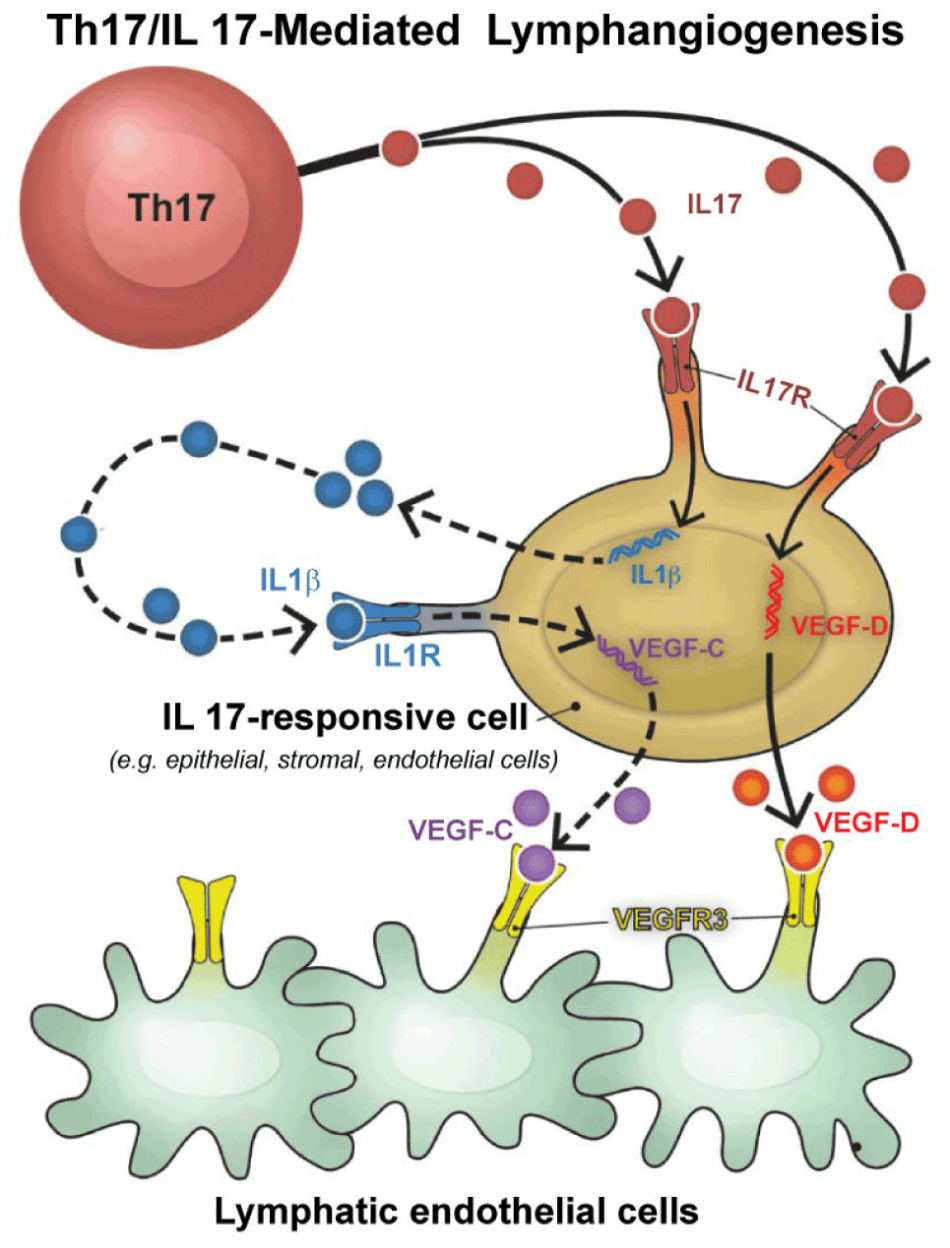
Mechanism of T-helper-17 (Th17) cell-mediated lymphangiogenesis. Th17 cell-secreted interleukin-17 (IL-17) induces lymphangiogenesis via a vascular endothelial growth factor (VEGF)-D/C–VEGFR-3 signaling pathway. In response to Th17-secreted IL-17, IL-17-receptor-expressing cells such as epithelial and stromal cells directly upregulate expression of VEGF-D; whereas VEGF-C expression is upregulated via IL-17-induced IL-1β-mediated pathway. Both VEGF-D and VEGF-C bind to VEGFR-3 on lymphatic endothelial cells and promote proliferation and tube formation by lymphatic endothelial cells.

Our studies of the cornea in dry eyes have revealed that these corneas significantly up-regulate pro-lymphangiogenic VEGF-C and VEGF-D along with their receptor VEGFR-3, suggesting that the low-grade chronic inflammation seen in dry eyes is selectively conducive for lymphangiogenesis. Th17 cells, in addition to their proinflammatory functions, have been recognized as potent inducers of angiogenesis in autoimmune diseases and malignancies [79,80], but little was known about their function as inducers of lymphatic growth. In dry eye disease Th17 cell-secreted IL-17 promotes a selective ingrowth of new corneal lymphatic vessels (Figure 4) by inducing increased expression of pro-lymphangiogenic VEGF-D and VEGF-C by epithelial, stromal and resident immune cells in the cornea that can induce proliferation of lymphatic endothelial cells [80]. Importantly, we showed that in vivo blockade of IL-17 in dry eye disease significantly reduces corneal lymphangiogenesis and the progression of clinical disease. Taken together, these findings suggest that in addition to causing corneal damage, Th17 cell-secreted IL-17 promotes the growth of corneal lymphatic vessels in autoimmune dry eye disease. Despite the fact that innate immunity plays a crucial role in inducing lymphangiogenesis, ingrowth of lymphatic vessels in dry eye corneas indicate a new adaptive immune Th17/IL17-mediated mechanism in inducing lymphangiogenesis.

## Conclusions

The absence of lymphatic vessels contributes significantly to the immune privileged state of the cornea by blunting ocular antigen and APC trafficking to the draining lymph nodes, and thus the induction of adaptive immunity. Injury and inflammation, however, can induce lymphangiogenesis and result in loss of immune privilege, allowing immune responses in the cornea. Complex cellular and molecular mechanisms regulate lymphangiogenesis in diverse corneal inflammatory conditions, and only recently, data provide evidence for important functional interactions between corneal lymphangiogenesis and the adaptive immune response in autoimmunity. In most corneal inflammatory diseases lymphangiogenesis is closely linked to hemangiogenesis—together they serve as major routes of induction and expression of adaptive immunity such as alloimmune response, which is critical in transplant rejection. However, in chronic autoimmune ocular surface inflammation such as dry eye disease, there is an exclusive growth of corneal lymphatics (without blood vessels), which primarily occurs in response to a Th17-mediated autoimmune response. Last, the cornea serves as an ideal site for angiogenic studies (as well as vascular endothelial cell-immune cell interactions) due to its accessible location, transparent nature, and its blood vessel- and lymphatic-free character. Understanding the mechanisms underlying corneal lymphangiogenesis will likely aid in developing more specific therapeutic strategies of broader clinical conditions beyond the treatment of corneal inflammatory conditions alone.
